# Observation of Rayleigh-Lamb waves generated by the 2022 Hunga-Tonga volcanic eruption with the POLA detectors at Ny-Ålesund

**DOI:** 10.1038/s41598-022-23984-2

**Published:** 2022-11-20

**Authors:** M. Abbrescia, C. Avanzini, L. Baldini, R. Baldini Ferroli, G. Batignani, M. Battaglieri, S. Boi, E. Bossini, F. Carnesecchi, M. Casula, D. Cavazza, C. Cicalò, L. Cifarelli, F. Coccetti, E. Coccia, A. Corvaglia, D. De Gruttola, S. De Pasquale, L. Galante, M. Garbini, G. Gemme, I. Gnesi, E. Gramstad, S. Grazzi, E. S. Haland, D. Hatzifotiadou, P. La Rocca, Z. Liu, L. Lombardo, G. Mandaglio, A. Margotti, G. Maron, M. N. Mazziotta, M. Mazzola, A. Mulliri, R. Nania, F. Noferini, F. Nozzoli, F. Ould-Saada, F. Palmonari, M. Panareo, M. P. Panetta, R. Paoletti, M. Parvis, C. Pellegrino, L. Perasso, O. Pinazza, C. Pinto, S. Pisano, F. Riggi, G. Righini, C. Ripoli, M. Rizzi, G. Sartorelli, E. Scapparone, M. Schioppa, G. Scioli, A. Scribano, M. Selvi, M. Taiuti, G. Terreni, A. Trifirò, M. Trimarchi, A. P. Viola, C. Vistoli, L. Votano, M. C. S. Williams, A. Zichichi, R. Zuyeuski

**Affiliations:** 1 Dipartimento di Fisica “M. Merlin” dell’Università e del Politecnico di Bari, Via Amendola 173, 70125 Bari, Italy; 2 INFN, Sezione di Bari, via Orabona 4, 70126 Bari, Italy; 3 INFN, Sezione di Pisa, Largo Bruno Pontecorvo 3, 56127 Pisa, Italy; 4 Dipartimento di Fisica “E. Fermi”, Università di Pisa, Largo Bruno Pontecorvo 3, 56127 Pisa, Italy; 5 INFN, Laboratori Nazionali di Frascati, Via Enrico Fermi 54, 00044 Frascati, RM Italy; 6 INFN, Sezione di Genova, Via Dodecaneso, 33, 16146 Genova, Italy; 7 Dipartimento di Fisica, Università di Cagliari, S.P. Monserrato-Sestu Km 0,700, 09042 Monserrato, CA Italy; 8 INFN, Sezione di Cagliari, Complesso Universitario di Monserrato, S.P. per Sestu - Km 0,700, 09042 Monserrato, CA Italy; 9 CERN, Esplanade des Particules 1, 1211 Geneva 23, Switzerland; 10 Istituto di Scienze Polari - CNR sede di Venezia, Via Torino, 155, Venezia Mestre, VE Italy; 11 INFN, Sezione di Bologna, Viale Carlo Berti Pichat 6/2, 40127 Bologna, Italy; 12 Dipartimento di Fisica e Astronomia “A. Righi”, Università di Bologna, Viale Carlo Berti Pichat 6/2, 40127 Bologna, Italy; 13 Museo Storico della Fisica e Centro Studi e Ricerche “E. Fermi”, Via Panisperna 89/a, 00184 Rome, Italy; 14 Gran Sasso Science Institute, Viale Francesco Crispi 7, 67100 L’Aquila, Italy; 15 INFN, Sezione di Lecce, Via per Arnesano, 73100 Lecce, Italy; 16 Dipartimento di Fisica “E. R. Caianiello”, Università di Salerno, Via Giovanni Paolo II, 132, 84084 Fisciano, SA Italy; 17 INFN, Gruppo Collegato di Salerno, Complesso Universitario di Monte S. Angelo ed. 6, Via Cintia, 80126 Naples, Italy; 18 Teaching and Language Lab (TLLab), Politecnico di Torino, Corso Duca degli Abruzzi 24, Turin, Italy; 19 INFN, Gruppo Collegato di Cosenza, Via Pietro Bucci, Rende, Cosenza Italy; 20 Physics Department, Oslo University, P.O. Box 1048, 0316 Oslo, Norway; 21 Dipartimento di Scienze Matematiche e Informatiche, Scienze Fisiche e Scienze della Terra, Università di Messina, Viale Ferdinando Stagno d’Alcontres 31, 98166 Messina, ME Italy; 22 Dipartimento di Fisica “E. Majorana”, Università degli Studi di Catania, Via S. Sofia 64, 95123 Catania, Italy; 23 INFN, Sezione di Catania, Via S. Sofia 64, 95123 Catania, Italy; 24 ICSC World laboratory, Geneva, Switzerland; 25 Dipartimento di Elettronica e Telecomunicazioni, Politecnico di Torino, corso Duca degli Abruzzi 24, Turin, Italy; 26 INFN, Laboratori Nazionali di Legnaro, Viale dell’Università 2, 35020 Legnaro, Italy; 27 Istituto di Scienze Polari - CNR Area della ricerca di Bologna, Via Piero Gobetti 101, Bologna, Italy; 28 INFN Trento Institute for Fundamental Physics and Applications, Via Sommarive, 14, 38123 Povo, TN Italy; 29 Dipartimento di Matematica e Fisica “E. De Giorgi”, Università del Salento, Via per Arnesano, 73100 Lecce, Italy; 30 Dipartimento di Scienze Fisiche, della Terra e dell’Ambiente, Università di Siena, Via Roma 56, 53100 Siena, Italy; 31 INFN-CNAF, Viale Carlo Berti PIchat 6/2, 40127 Bologna, Italy; 32 Physik Department, Technische Universitat Munchen, James-Franck-Straße 1, 85748 Garching bei München, Germany; 33 CNR Istituto di Fisica Applicata “Nello Carrara”, Via Madonna del Piano 10, 50019 Sesto Fiorentino, FI Italy; 34 Dipartimento di Fisica, Università della Calabria, Via Pietro Bucci, Rende, CS Italy; 35 Dipartimento di Fisica, Università di Genova, Via Dodecaneso, 33, 16146 Genova, Italy; 36 Istituto di Scienze Polari - CNR Area della ricerca di Roma Tor Vergata, Via Fosso del Cavaliere 100, Rome, Italy; 37 INFN, Laboratori Nazionali del Gran Sasso, Via G. Acitelli 22, 67100 Assergi, AQ Italy

**Keywords:** Techniques and instrumentation, Volcanology

## Abstract

The eruption of the Hunga-Tonga volcano in the South Pacific Ocean on January 15, 2022, at about 4:15 UTC, generated a violent explosion, which created atmospheric pressure disturbances in the form of Rayleigh-Lamb waves detected all over the globe. Here we discuss the observation of the Hunga-Tonga shock-wave performed at the Ny-Ålesund Research Station on the Spitsbergen island, by the detectors of the PolarquEEEst experiment and their ancillary sensors. Online pressure data as well as the results of dedicated offline analysis are presented and discussed in details. Results include wave arrival times, wave amplitude measurements and wave velocity calculation. We observed five passages of the shock wave with a significance larger than 3 $\sigma$ and an amplitude up to 1 hPa. The average propagation velocity resulted to be (308 ± 0.6) m/s. Possible effects of the atmospheric pressure variation associated with the shock-wave multiple passages on the cosmic-ray rate at ground level are also investigated. We did not find any significant evidence of this effect.

## Introduction

On December 20, 2021, an eruption began on Hunga Tonga-Hunga Ha’apai, an uninhabited island of the Tongan archipelago, located in the southern Pacific Ocean, where a submarine volcano exists. After a period of activity of variable intensity, the eruption resumed on January 14, 2022 at 4:20 local time^1^. The following day, January 15, 2022, at 17:14:45 local time, corresponding to 04:14:45 UTC, an extremely violent explosion took place, destroying part of the island, sending clouds of ash 40 km high into the atmosphere, causing tsunamis in Tonga, Fiji, American Samoa, Vanuatu, and along the Pacific rim^2^.

This explosion was heard in various parts of the world, like Samoa, New Zealand (more than 2000 km away from the eruption point), and, in the form of a series of bangs, even as far as Alaska^3^. Also, an atmospheric shock-wave propagated around the globe, whose associated pressure disturbances were measured by weather stations at many locations^2,4^. For instance, in New Zealand the maximum amplitude of about 7 hPa was recorded. In Europe, the shock-wave was measured at various locations, with a typical amplitude in the 1$\div$3 hPa range^5^.

Very interestingly, there has been reports that the shock-wave has gone around the Earth two or even more times. It is relatively rare for a volcano to generate a shock-wave strong enough to circle many times the globe. Another famous eruption giving rise to similar effects was the one of the Krakatoa volcano in Indonesia, which occurred in 1883^6^ and was recorded by barometers all around the world, and is now considered a pioneering event in infra-sound monitoring studies. Consequently, there is an intrinsic interest for all the data about the Hunga Tonga eruption, and the revolutions around the Earth of its associated shock-wave.

Here we report about the observation of the Hunga Tonga shock-wave performed by the detectors of the PolarquEEEst experiment (indicated hereafter as “POLA detectors”, for brevity) located at the Ny-Ålesund Research Station on the Spitsbergen island, in the Svalbard archipelago, at 78$^{\circ }$55’30”N - 11$^{\circ }$55’20”E, less than one thousand kilometers from the North Pole^7^.

The pressure data presented here are original and they can be added to the worldwide set of data currently available. They are particularly interesting since they have been collected close to the North Pole, far away from the usual public stations. For instance, most of the observations reported in the current literature^4,8,9^ are from USA and Europe, therefore they are distributed on the globe quite disuniformly. Moreover, the measurement presented in this paper allow to investigate a different propagation direction from the eruption point, improving the multi-technology observations of the event.

The POLA detectors were conceived for long term monitoring of secondary cosmic rays at sea level, and have been in operation for almost three years now. The scientific program of the PolarquEEEst experiment includes the search for possible correlations between the cosmic rate measured at high latitudes with environmental variables, either local (atmospheric), or cosmic (space weather), a topic only partially studied up to now. Therefore, the POLA detectors are equipped with multiple sensors for monitoring in real time the atmospheric pressure, temperature and humidity, and the local magnetic and gravitational fields, that provided the possibility to record the shock-wave from the Hunga Tonga volcano with a precision similar than most measurements reported up to now.

A study correlating the observations performed by the POLA sensors and the others performed at various locations in the world, in terms of arrival times, amplitude and other observable characteristics of the shock-wave, with a model describing the propagation in the atmosphere of the shock-wave, could provide essential information for the definition of the parameters of these models. We are aware of one study, reported in Reference^4^. Therefore, providing experimental data is of utmost importance.

## Results

### Real-time data analysis

**Figure 1:**
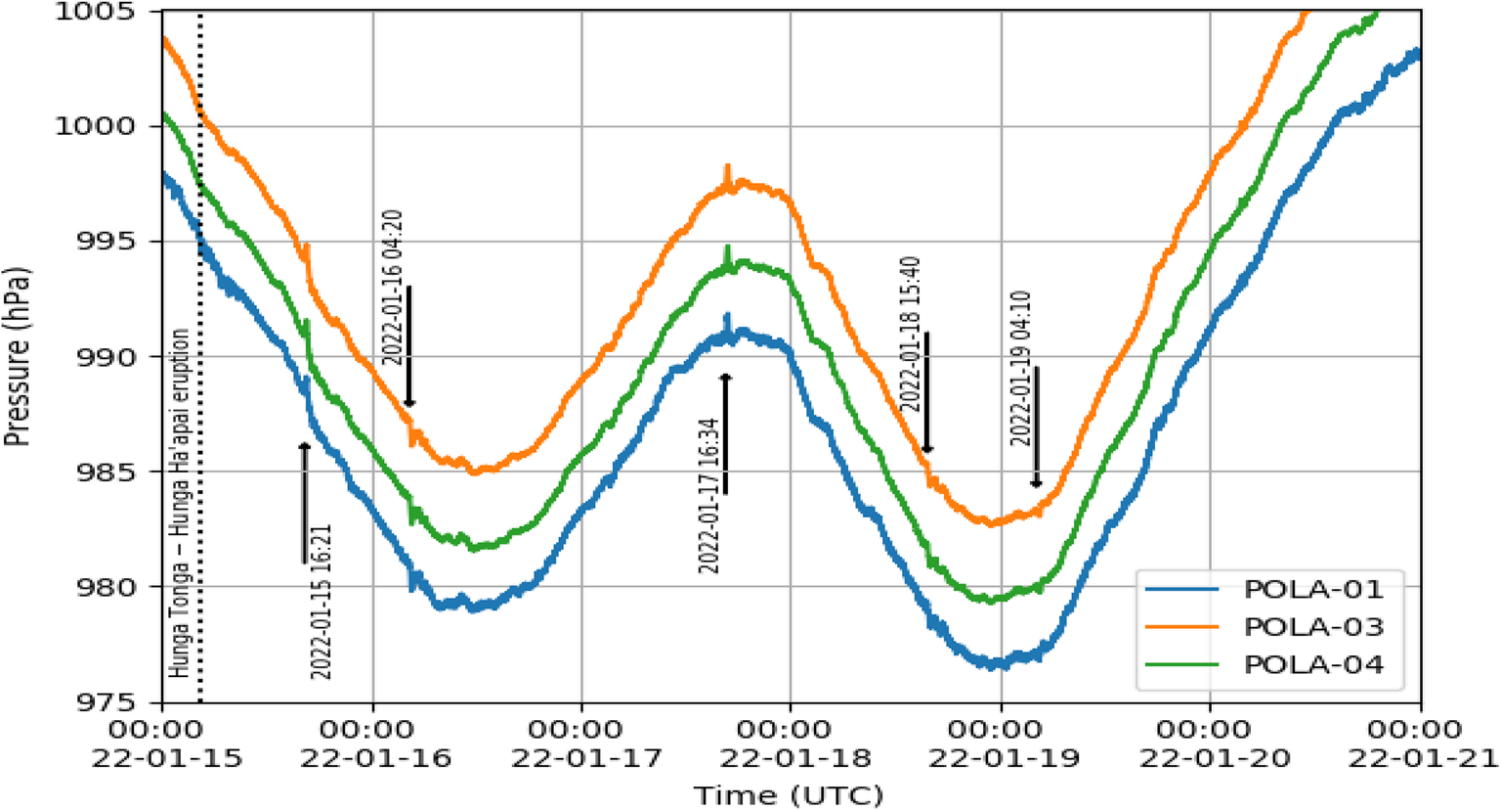
Real time pressure as a function of time (UTC) from January 15 to January 21, 2022 observed with POLA-01 (blue line), POLA-03 (orange line) and POLA-04 (green line), respectively.

During the usual online monitoring of the data of the POLA detectors located at Ny-Ålesund, and prompted by the news of the Hunga-Tonga volcano eruption, we carefully checked the real time pressure and rate measurements. The pressure readouts, taken in time steps of 30 seconds, for a period of 6 days starting from January 15, 2022, are shown in Fig. 1.

**Figure 2:**
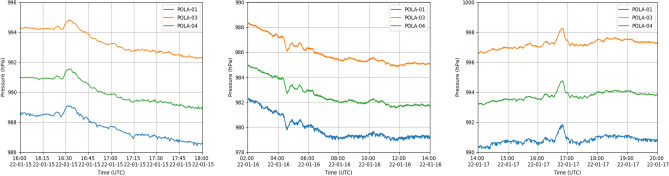
Real time pressure as a function of time of the first three main pressure features observed with POLA-01 (blue line), POLA-03 (orange line) and POLA-04 (green line), respectively. Left panel: pressure feature observed on January 15, 2022; Central panel: pressure feature observed on January 16, 2022; Right panel: pressure feature observed on January 17, 2022.

Two different pressure sensor models are installed on the POLA detectors, namely LPS25H^10^ and BME280^11,12^. Only data from the LPS25H sensors are shown in Fig. 1, and thereafter, while the BME280 sensor data were only used as cross check.

The pressure values measured at the same time by the three POLA detectors shown in Fig. 1 are characterized by some small absolute differences across each other, which we will hereafter call “offsets”. These are explained, for the most part, by the different altitudes the three POLA detectors were located at Ny-Ålesund, namely 11 m, 43 m and 52 m a.s.l, for POLA-03, POLA-04 and POLA-01, respectively. The residual offsets can be attributed to slight differences in construction and mis-calibration across the devices. Since we are interested in pressure variations around the baseline, the presence of these offsets has no effects on the results discussed here. Moreover, during our laboratory tests, before the installation at Ny-Ålesund, random fluctuations around the baseline were measured to be less than about 0.1 hPa (RMS). Moreover, from the datasheet of the LPS25H^10^ pressure sensor, the noise RMS is quoted about 0.01–0.03 hPa, while the BME280^11^ is 0.2Pa RMS.

Indeed, some sharp pressure variations are well visible by eye, corresponding to various passages of the atmospheric shock wave through the Ny-Ålesund site. Close-ups taken around the detection times of the first three pressure features observed are shown in Fig. 2.

The first pressure feature was detected on January 15, and started at about 16:21 UTC, then reached a maximum of roughly +0.7 hPa (an overpressure) above the baseline at 16:33 UTC, about 12 h and 18 min after the volcano violent explosion, and we believe it corresponds to the first passage of the shock-wave. Roughly speaking, the duration of this main peak associated to the perturbation is about 10 min. Moreover, the maximum is clearly seen to be preceded by a precursor, of much lower intensity, well separated from the main peak, and a barely visible successor, about 45 min after the maximum, of negative intensity.

Estimating as 13,519 km the distance between Ny-Ålesund and Hunga-Tonga, the average transit velocity of the wave can be deduced to be about ($305.0 \pm 1.5$) m/s, if one assumes that the maximum of the pressure perturbation corresponds to the climax of the explosion. We computed the distance between the two locations along the surface of the Earth, according to what reported in Ref.^13^, namely approximating the Earth geoid with a sphere of 6372.795 km radius. This approximation gives origin to an error on the distance computed of around 0.3%, which is reflected in a ±1 m/s error on the computed velocity. The error due to the uncertainty about the time of climax of the eruption and the time of the passage of the shock-wave, estimated to be a couple of minutes altogether, contributes for another 1 m/s, to be added in quadrature to the uncertainty discussed above.

In the calculations presented here we assume that the detected shock-wave travels very close to the surface of the Earth, since we do not have any direct information about at which effective altitude it travelled. Of course, the effective altitude at which the shock wave actually propagates affects the distance travelled, and therefore the computed velocity. However, the expected associated systematic error is smaller than the ones previously cited.

A second pressure anomaly was detected to start on January 16 at about 4:20 UTC, namely 24 h and 5 min after the eruption, with a negative sharp fluctuation with respect to the baseline, reaching a maximum of about 0.7 hPa about 16 min later. We assume that this is the passage of another part of the shock wave, travelling in the opposite direction and passing through the antipode, and therefore covering a total distance of about 26522 km, corresponding to an average velocity of about ($302.4 \pm 0.7$) m/s. Given the longer distance and time travelled, the estimated velocity is characterized by a smaller error than the one computed for the first pressure feature. This perturbation appears to be preceded by two smaller precursors, with negative pressure fluctuations with respect to the baseline, and two successors, positive with respect to the baseline. Also two other successors are visible, about 45 min after the minimum pressure value, positive with respect to the baseline.

The third pressure perturbation was detected on January 17, with a positive maximum with respect to the baseline of about 0.7 hPa measured at 16:47 UTC, 60 h and 27 min after the eruption. We believe that this is the second passage of the first detected shock-wave already detected on January 16, at 4:20 UTC, after turning around the world, namely travelling a total distance of about 66525 km. It is preceded by two precursors and followed by two successors about 70 min later. The measured shock wave average velocity results to be ($305.9 \pm 0.4$) m/s, in good agreement with the previous results. Both second and third perturbation appear to have a duration of around 15 min.

Apparently, the second passage of the shock-wave, first detected on January 15 and predicted to re-appear at around 5 UTC of January 17 (assuming a velocity of 305 m/s) is not visible in the data. Two further pressure features were detected due to subsequent passages of the shock wave across Ny-Ålesund: one on January 18, at 15:40 UTC, and the other on January 19, at 04:10, both characterized by a significantly smaller amplitude with respect to the previous ones.

**Figure 3:**
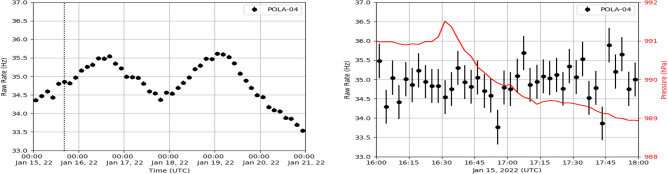
Left panel: POLA-04 3-h raw particle rate as a function of time (UTC), from January 15 to January 21, 2022. The vertical dotted line corresponds to the time of the first observed pressure anomaly. Right panel: 3-min POLA-04 particle rate (black point, left axis) and pressure (red line, right axis) observed on January 15, from 16:00 to 18:00 UTC.

While measuring the atmospheric pressure variations, the POLA detector continued to take data on the charged cosmic particle rate. The rate measured by the POLA-04 detector, in bins of 3 h, from January 15 until January 21, 2022 is shown in Fig.  3 (left panel). The same particle rate, measured in time intervals of 3 min, is shown as black dots in the right panel of Fig. 3 together with the pressure values, shown in red, measured on January 15, namely during the passage of the first shock wave.

In principle, the cosmic particle rate is expected to be influenced by atmospheric pressure variations, since these change the average height of the primary interaction on top of the atmosphere and the density of the medium the subsequent Extensive Atmospheric Shower propagates through. This so called “barometric” effect predicts an inverse correlation between cosmic particle rate measured on the ground and atmospheric pressure, and has been experimentally checked with the POLA detectors^7^. In Fig. 3, to put in evidence a possible effect on cosmic particle rate as a result of the passage of the shock-wave, the raw particle rate, namely not corrected for the pressure, is shown.

The rate observed by the POLA-04 detector and shown in the Fig. 3 does not show variations, correlated with the observed pressure feature, significantly larger than the usual expected statistical fluctuations. The plots obtained with the data from POLA-01 and POLA-03, not reported here, lead to similar conclusions. Note that, in order to reduce the statistical fluctuations, one should perform an analysis with much larger time bins, but in that case the resolution in time needed to correlate cosmic charged particle rate variations with the pressure feature would be lost. We also performed an analysis combining the data from the three POLA detectors, but no significant variation of the particle rate associated to the shock-wave was observed.

Indeed, due to the limited acceptance of the POLA detectors and the fact that the rate variation with pressure is expected to be about a few thousandths of Hz per hPa^7^, the POLA detectors are not foreseen to be able observe any significant (i.e. larger than statistical fluctuations) change in the cosmic particle rate correlated with the passage of the shock-wave through Ny-Ålesund.

**Figure 4:**
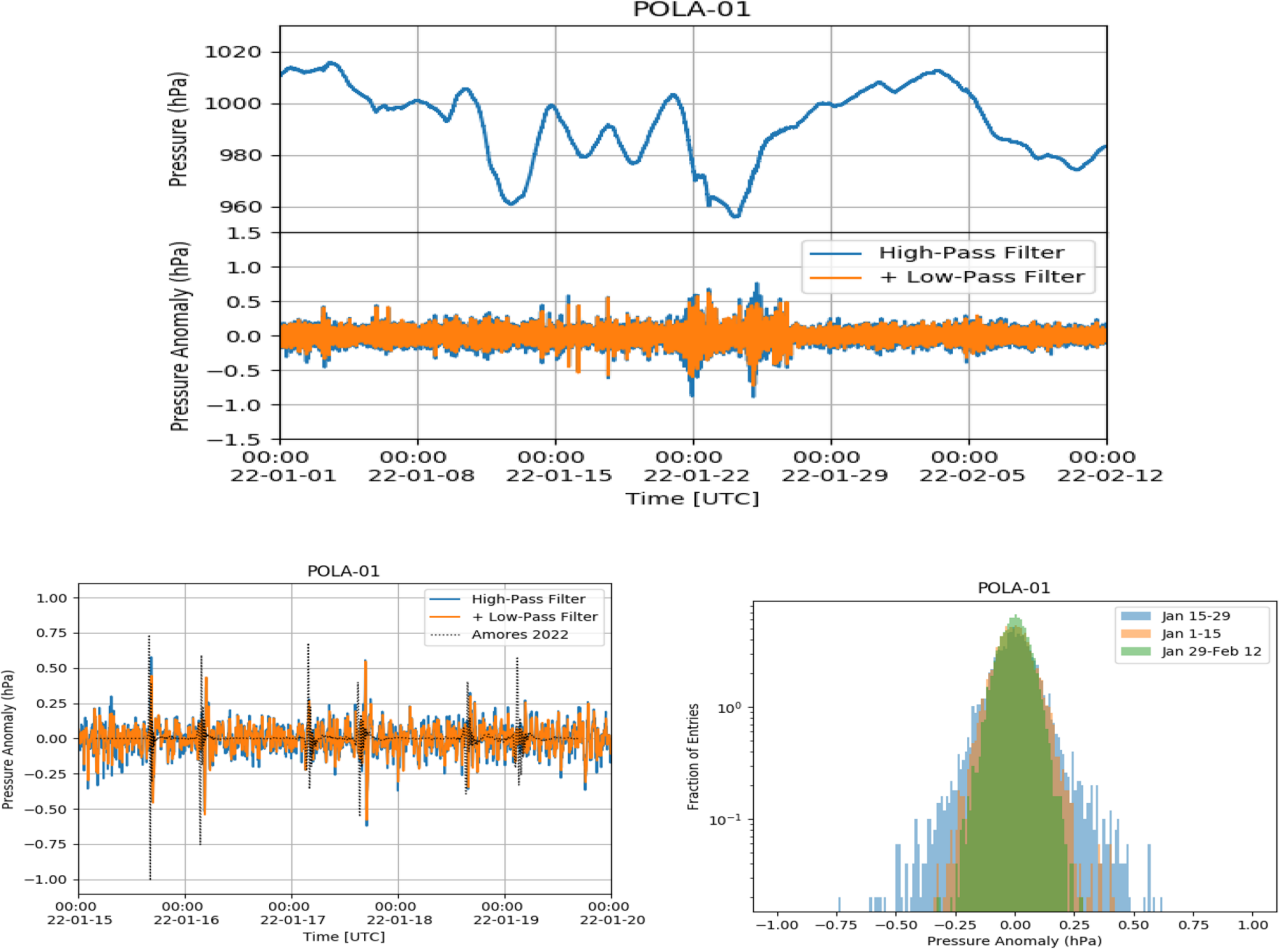
Top: POLA-01 pressure (top panel) and pressure anomaly (bottom panel) from January 1 until February 12. Bottom left: zoom of pressure anomaly from January 15 to January 20. The dotted line corresponds to the simulation data of Ref.^4^. Bottom right: pressure anomaly distribution from January 15 to January 29 (blue area), from January 1 to January 15 (orange area) and from January 29 to February 12 (green area), respectively.

**Figure 5:**
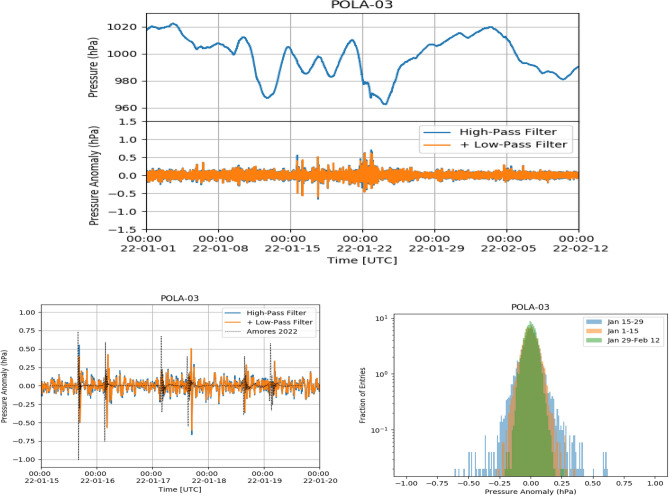
Top: POLA-03 pressure (top panel) and pressure anomaly (bottom panel) from January 1 until February 12. Bottom left: zoom of pressure anomaly from January 15 to January 20. The dotted line corresponds to the simulation data of Ref.^4^. Bottom right: pressure anomaly distribution from January 15 to January 29 (blue area), from January 1 to January 15 (orange area) and from January 29 to February 12 (green area), respectively.

**Figure 6:**
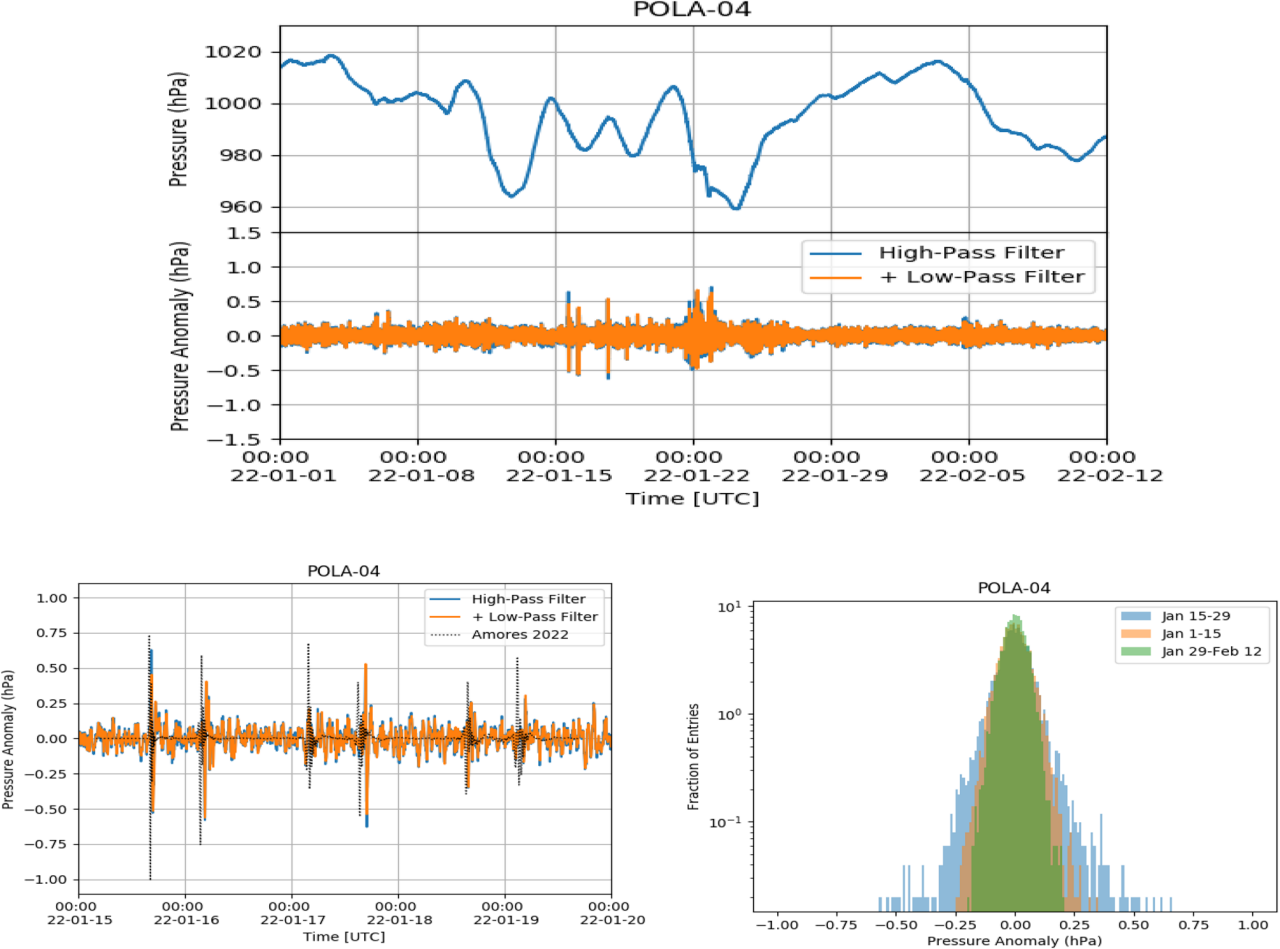
Top: POLA-04 pressure (top panel) and pressure anomaly (bottom panel) from January 1 until February 12. Bottom left: zoom of pressure anomaly from January 15 to January 20. The dotted line corresponds to the simulation data of Ref.^4^. Bottom right: pressure anomaly distribution from January 15 to January 29 (blue area), from January 1 to January 15 (orange area) and from January 29 to February 12 (green area), respectively.

**Figure 7:**
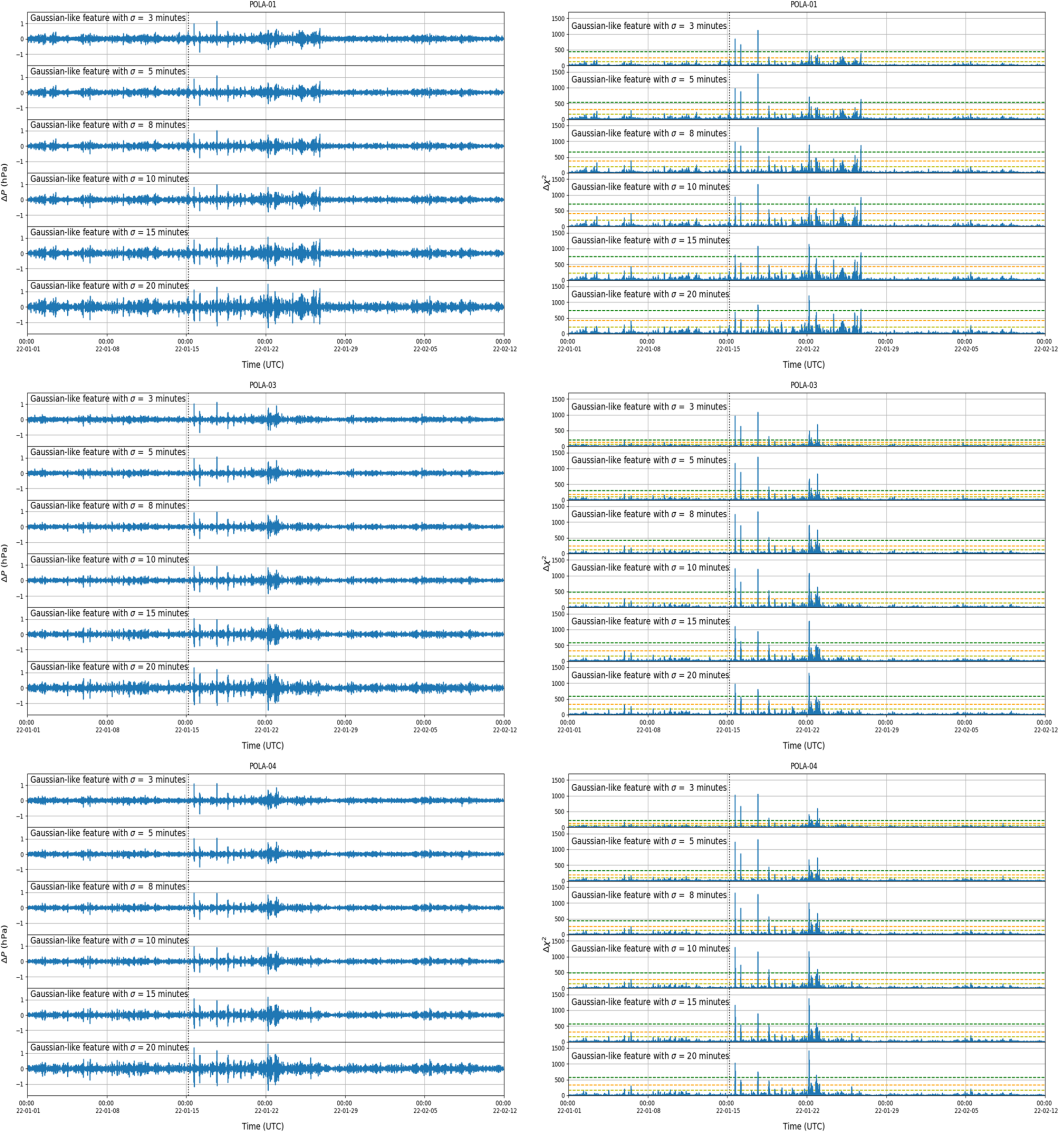
POLA-01 (top), POLA-03 (middle) and POLA-04 (bottom) pressure feature (left) and local significance (right) as a function of time. The Gaussian-like feature has a fixed sigma (from top to bottom) of 3, 5, 8, 10, 15 and 20 min, respectively. The yellow, orange and green horizontal lines show the 3, 4 and 5 global significance sigma, respectively. The vertical dotted line corresponds to the eruption time.

### Offline data analysis results

In order to better understand the relation between the Hunga-Tonga volcano event and the data collected by the POLA detectors, we also performed two independent dedicated offline data analysis, designed to search for all possible pressure variations in a time range of several weeks, and related to the atmospheric shock-wave generated by the eruption. The purpose of extending the data analysis to the data collected up to mid-February was to look for possible additional pressure anomalies that were not detected in the first rapid analysis, possibly related to the shock-wave travelling once more around the Earth.

In the first analysis we filtered the pressure data with a band-pass filter, in order to search for pressure anomalies in the data, characterized to be in a specific frequency range. In the second, we used a sophisticated fit procedure, often employed to look for small signals against a variable background, to search for short time features in the pressure measurements. Basically, it consists in fitting the rapid varying sought-for signal with a Gaussian function of amplitude $\Delta P$ and width $\sigma$, positioned over a second-order polynomial representing the natural smooth pressure variations. Both methods are described in details in the section “Materials and methods” of the present paper.

The atmospheric pressure measured with the POLA-01, POLA-03 and POLA-04 detectors, as a function of time from January 1 to February 15, 2022, together with the same data to which the filtering procedure cited above and discussed in the following was applied, are shown in Figs. 4, 5 and 6, respectively. Filtered data are shown both after the first step, when frequencies lower than 1/7200 Hz are filtered out, and after the second, when also frequencies higher than 1/900 Hz are cut. Actually, as can be inferred from the above figures, the second filter has a minor effect on the global result. We call pressure data, after the band-pass filter was applied, pressure “anomaly”.

Given the 30 s time bin and the one month time span used for the analysis, the pressure shock-waves present in Fig. 1 are no more visible by eye in their upper panel. However, starting from January 15, some spikes are clearly visible in the filtered data, in all three detectors, corresponding to the various passages of the Hunga-Tonga shock-wave. After around January 24, the peak-to-peak fluctuations are similar to the ones observed before January 15, and no relevant structure can be spotted. It is interesting to note that also some broad two-peak pressure anomaly structures can be observed, starting just after the midnight UTC of January 22. If this is due to further passages of the shock-wave, it means that the volcano created a pressure shock still significant even after one week after its eruption, and this would be quite significant. While data from all detectors show the same basic behaviour, the POLA-01 pressure sensor shows slightly larger average peak-to-peak fluctuations than POLA-03 and POLA-04 ones. For the sake of clarity, zooms of the pressure anomalies are also shown in the bottom left panels of the same Figs. 4,  5 and  6, and one can check that the times corresponding to the maxima of its module approximately match the ones estimated looking at the plot in Fig. 1, and reported above.

In the same bottom left panels, we have reported the predictions of the 5-days model for the arrival times of the shock waves at Ny-Ålesund taken from Ref.^4^. Characteristics of the model, which, in general, show a nice agreement with the data, is that it predicts a two-peak structure, which is the one actually observed. However, there is an around 30 min delay between the predicted arrival time of the first shock-wave, with respect to the observed one. This progressively increases for the subsequent passages. Also, the predicted amplitude of the pressure anomaly and its polarity are slightly different with respect the observed one. The third passage of the wave, in the morning of January 17, is predicted to be comparable in intensity to the previous two ones, but is barely significant in our data, even if the sensitivity of our instrumentation (made of three independent devices, each equipped with two different pressure sensors) would in principle be able to clearly detect it, like the others were detected. Also at other locations (like the pressure sensors located at Manhattan, or North Adams), the passage of the shock-wave was not detected so clearly^4^.

The pressure anomaly amplitude distributions, measured from January 15–29, from January 1 to January 15 and from January 29 to February 12, are shown, in different colors, in the bottom right panels of the same Figs. 4,  5 and  6. From their examination one can deduce that, as expected, the largest fluctuations in the pressure data after filtering are observed in the first of the three periods cited above, while before it (namely, in the control zone) and after it, when the perturbation has already passed, are smaller and comparable.

The pressure feature $\Delta P$, calculated with the Gaussian fitting procedure previously cited and discussed later, is shown in left top panel of Fig. 7, for the POLA-01, POLA-03 and POLA-04 detectors, respectively. Results obtained with values for $\sigma$ of 3, 5, 8, 10, 15 and 20 min are shown separately. Again, starting from January 15, some significant features are observed in the pressure measurements taken by all three POLA detectors. The corresponding $\Delta \chi ^2$ of the fit is also shown in the right panel of the same figure, and compared with the values of the global significance $\sigma _{global}$ of the fit, giving an estimate of the probability that a signal is indeed present. The first three pressure features are clearly visible, with $\Delta \chi ^2$ values almost always larger than five times the local significance. Moreover, the largest values of $\Delta \chi ^2$ appear when values for $\sigma$ of 5 or 8 min are chosen; this is reasonable and coherent with the time duration of the pressure features observed during the online analysis of the data.

Again, significant pressure features appear around just after the midnight UTC of January 22, corresponding to the features also observed with the filter analysis. They show a $\Delta \chi ^2$ larger than five times the local significance. The largest values of $\Delta \chi ^2$, in this case, are obtained when $\sigma$ = 20 min is chosen. This could be coherent with the fact that the perturbation, after having travelled for about one week in the atmosphere, was more spread in time, and its intensity was therefore reduced and could not be directly revealed with a simple online analysis.

**Figure 8:**
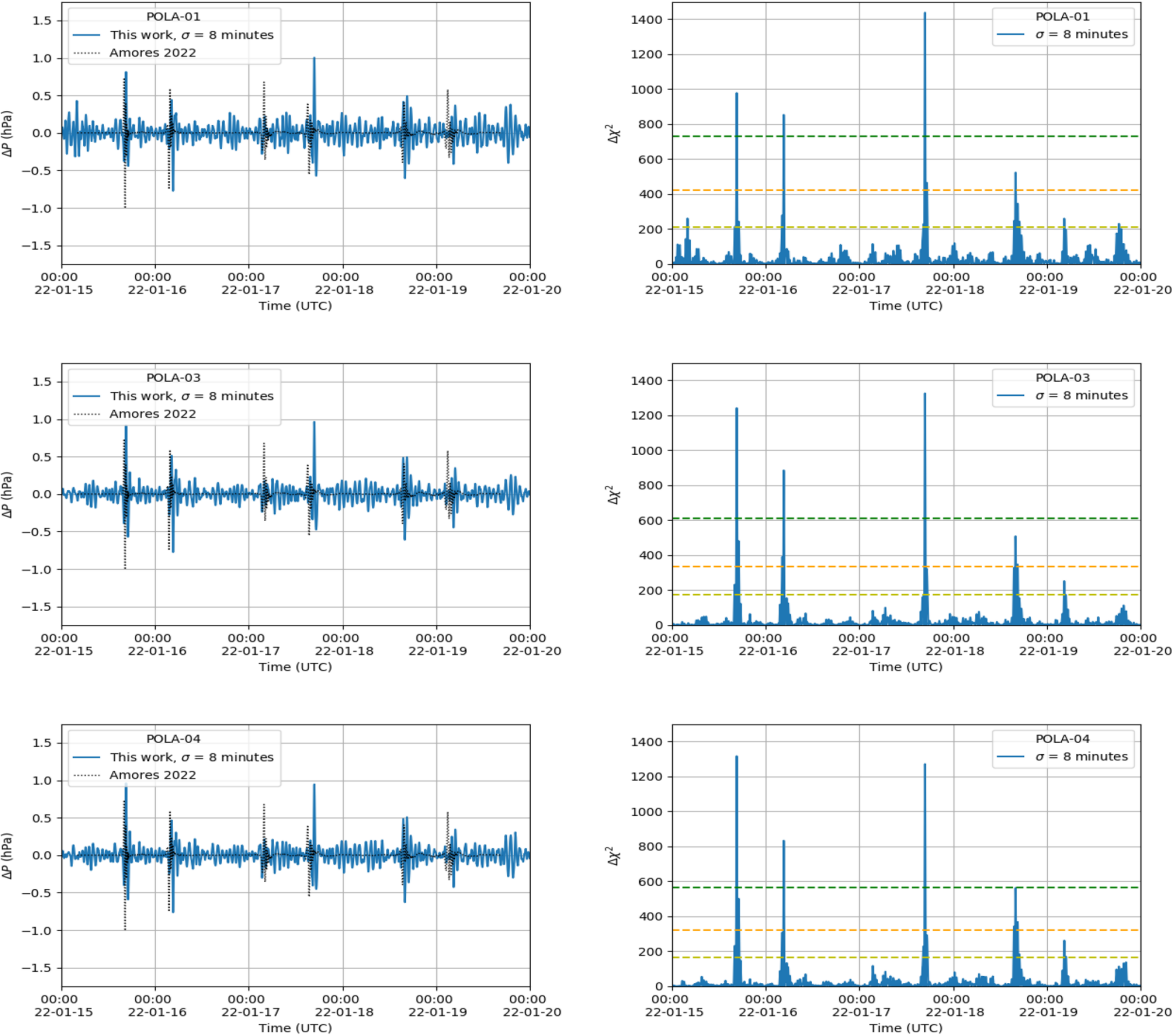
Pressure feature (left column) and significance (right column) from January 15 to January 20, measured with (top to bottom) POLA-01, POLA-03 and POLA-04 sensors, respectively. The Gaussian-like feature corresponds to $\sigma = 8$ min. The yellow, orange and green horizontal lines show the 3, 4 and 5 global significance sigma, respectively. The dotted line corresponds to the simulation data of Ref.^4^.

The results obtained with a Gaussian $\sigma =$ 8 min are summarized in Fig. 8 where we show the feature pressure amplitudes $\Delta P$ and the significances $\Delta \chi ^2$ measured by the POLA detectors in the time period from January 15 to January 20, when the shock-wave was surely detected. Those features have amplitudes up to ± 1 hPa, and $\Delta \chi ^2$ all larger than 3 $\sigma _{global}$, and often more than five times. Again, the POLA-01 pressure sensor shows a slightly larger peak-to-peak noise. The prediction of the above cited model discussed in Ref.^4^ are also superimposed, showing a nice agreement for what concerns times and amplitude of the five features observed. As already pointed out, the pressure feature foreseen for first hours of January 17 is hardly distinguishable, even with this analysis.

**Figure 9:**
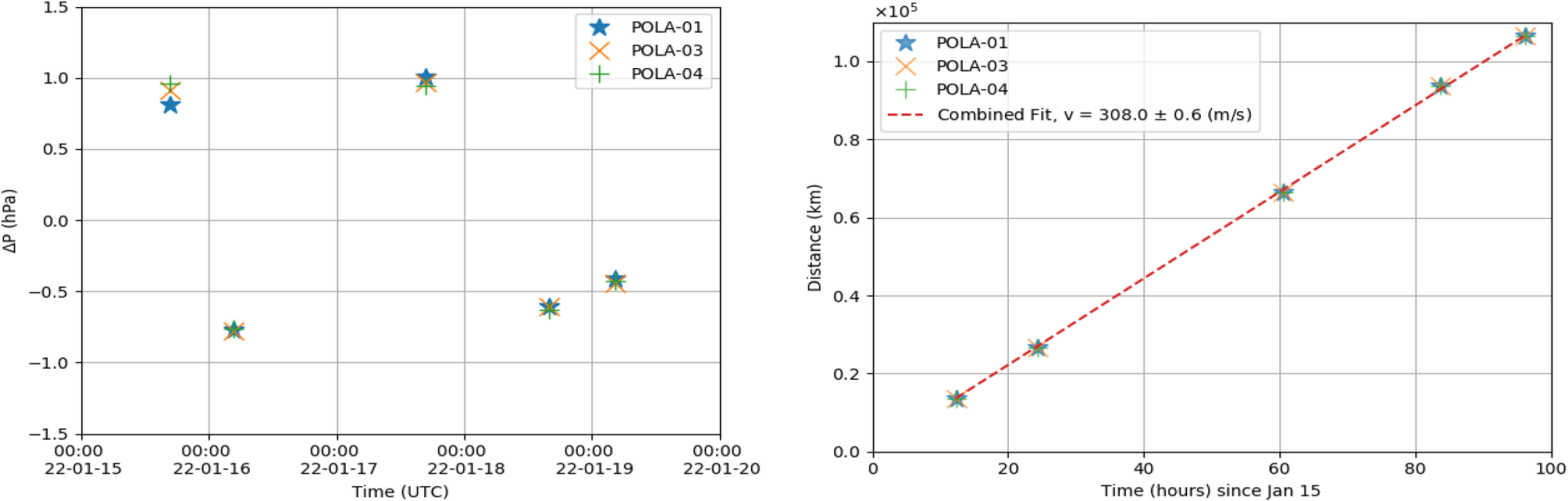
Most significant Gaussian-like pressure features with $\sigma$= 8 min calculated for the three detector sensors. Left panel: Most significant pressure anomalies as a function of the arrival time. Right panel: Total distance as a function of the arrival time. The average velocity combining all measurements is v = (308.0 ± 0.6) m/s. The error does not include the uncertainty on the distance.

The most significant Gaussian-like pressure anomalies amplitudes $\Delta P$ are shown in Fig. 9 as a function of the time they were detected. In this case, the value $\sigma$ = 8 min was chosen. As can be inferred from the figure, the first and the third passage of the shock waves created an increase of the pressure up to about 1 hPa, while the other passages created a decrease of the pressure of around about 0.5 $\div$ 0.7 hPa.

Shock-waves like the one generated by the Hunga-Tonga volcano are usually classified as Rayleigh-Lamb waves, since they propagate in the thin atmospheric layer wrapping the globe. Their amplitude at the detection point depends on the length of its approximately circular wave-front, and should be characterized by a minimum where the wave has travelled for a quarter of the Earth circumference, and by a maximum where the wave reaches the antipodean point of the eruption point. By energy conservation, denoting with $\Delta P_0$ the anomaly pressure corresponding to the volcano eruption, taking place over a cylinder of radius $r_0$, the pressure anomaly $\Delta P$ after the shock-wave has travelled a distance *d*, can be written as:

1 $$\begin{aligned} \Delta P = \Delta P_0 \sqrt{\frac{r_0}{2 \pi R \sin \left( \frac{d}{R} \right) }} \end{aligned}$$

where *R* is the radius of the Earth, assumed to the perfectly spherical. Of course, dissipative effects should be taken into account, which progressively reduce the shock-wave amplitude as a function of the distance travelled, and these are pretty evident in the subsequent passages of the shock-wave. A detailed discussion about the shock-wave amplitude and how it was influenced by the associated tsunami is reported in Ref.^2^.

**Table 1:** Most significant Gaussian-like pressure features with $\sigma$= 8 min calculated for the three detector sensors.

POLA-01	POLA-03	POLA-04	Distance (km)	Velocity (m/s)
2022-01-15 16:34:04	2022-01-15 16:34:05	2022-01-15 16:33:42	13519	304.9 ± 0.1
2022-01-16 04:37:05	2022-01-16 04:37:40	2022-01-16 04:37:19	26521	302.2 ± 0.1
2022-01-17 16:48:17	2022-01-17 16:47:19	2022-01-17 16:47:53	66561	305.3 ± 0.1
2022-01-18 16:00:48	2022-01-18 15:59:26	2022-01-18 16:00:02	93599	310.4 ± 0.1
2022-01-19 04:28:40	2022-01-19 04:27:59	2022-01-19 04:29:53	106601	307.7 ± 0.1

The total distance corresponding to each feature is reported and the average velocity is also calculated. The error does not include the uncertainty on the distance.

We summarize in Table 1 the detection times of the maxima of the most significant Gaussian-like pressure features, as computed with the best fit procedure, using $\sigma$= 8 min. The arrival times measured with the pressure sensors of the three detectors are in good agreement across each other, considered the 30 s time steps used in data acquisition, which reads the data independently from each POLA detector. The total distance travelled by the shock-wave corresponding to each feature is also reported in the same Table. It is computed in the same way as before^13^, taking into account the distance between Ny-Ålesund and the Hunga Tonga volcano. The average velocity of the shock-wave for each passage was also calculated and reported in the Table.

The total distance travelled by the shock-wave as a function of the detection time of the most significant Gaussian-like pressure features is shown in Fig. 9. Again $\sigma$= 8 min was used. Combining all measurements by performing a linear fit, the resulting average velocity v = (308.0 ± 0.6) m/s, where the error does not include the uncertainty on the distance. Another estimation of the Hunga Tonga shock-wave velocity, performed with analogous methods, is discussed in Ref.^14^, and the value found is 307 m/s, fully compatible with the present measurement. Also predictions reported in Ref.^15^ and our results are basically in agreement, once that experimental and theoretical uncertainties are properly taken into account.

The time when the shock-wave was recorded depends on the speed of the sound waves in air which, in turn, is influenced by many parameters, among which the most important is temperature. Also the presence of winds, and their direction, surely influenced the times the shock-wave was recorded in various parts of the globe. The measured velocity here results to be significantly lower than the sound speed in the atmosphere which is usually quoted for standard conditions (STP) and sea level, namely $\approx$ 343 m/s. This might be due to the fact that an important part of the pressure shock-wave travelled in the high atmosphere, where the lower temperature implies also a lower shock-wave velocity^16^.

Note that the prevailing winds along the route may be opposing or assisting, depending on if the considered part of the shock-wave was initially Northbound or Southbound, and this could influence the relative measured velocity. Experimental evidence for this effect was observed for the Hunga-Tonga volcano^17^, and even for the Krakatoa event^6^. The average velocity computed considering the first and fourth of the pressure anomalies of Table 1 (namely the ones approximately Southbound moving) is 307.6 m/s, while the one obtained computed considering the second, third and fifth of the same Table 1 (approximately Northbound moving) is 305.1 m/s.

## Conclusions

The shock-wave generated by the explosion of the Hunga Tonga-Hunga Ha’apai volcano was a once-in-a-hundred-years event, and the information that it will provide on the Earth-atmospheric system will prove to be invaluable. It has been sampled by multiple sensors, both on the ground and on satellites, differently from the Krakatoa event, which was even larger in magnitude but whose knowledge is limited by the instrumentation available at the time.

Here we have discussed detailed measurements of the shock-wave generated by the Hunga-Tonga volcano provided by the sensors hosted on the POLA detectors, which were originally conceived for astro-particle physics, and are collecting interesting data about secondary cosmic rays at a peculiar location, very close to the North Pole, where no other pressure sensors, to our knowledge, have reported similar data. Multiple passages of the shock wave were registered, and analysed by means of techniques well beyond the usual frequency analysis traditionally performed in this field, and exploiting algorithms that are employed to extract tiny signals over smooth background in multiple fields, from particle physics to neurology. This is an example of cross-contamination across different fields of the human knowledge.

Collected data were used to extract information about shock-wave amplitude and its arrival times, and from this about its group velocity, which turned out to be compatible both with theoretical predictions and other experimental measurements, performed at various locations in the world. Novelty is represented by the idea to investigate possible effects of the passage of the shock-wave on the cosmic particle rate measured at the ground level, which we could not be able to assess due to the low POLA detector acceptance, but whose existence was experimentally verified by another group exploiting essentially the same methodology presented here.

## Materials and methods

### The POLA detectors and sensors

The POLA detectors were developed by the Extreme Energy Events collaboration^18^, in the framework of the PolarquEEEst experiment. Goal of the PolarquEEEst experiment is to measure the cosmic ray rate at the sea level at the northernmost latitudes. Its first campaign of measurements took place in Summer 2018, when one of the POLA detectors was placed on board of a sailboat, named Nanuq, and whose results are presented in Ref.^7^.

In addition to the detector on board the sailboat, two other identical ones were assembled, and installed at two high schools located in Norway and in Italy, to be used for cross-reference. Those three PolarquEEEst detectors are called POLA-01, the one on board the sailboat Nanuq, while POLA-02 and POLA-03 were the ones located in Norway and Italy, respectively. A fourth detector was assembled in 2019 and labelled POLA-04.

**Figure 10:**
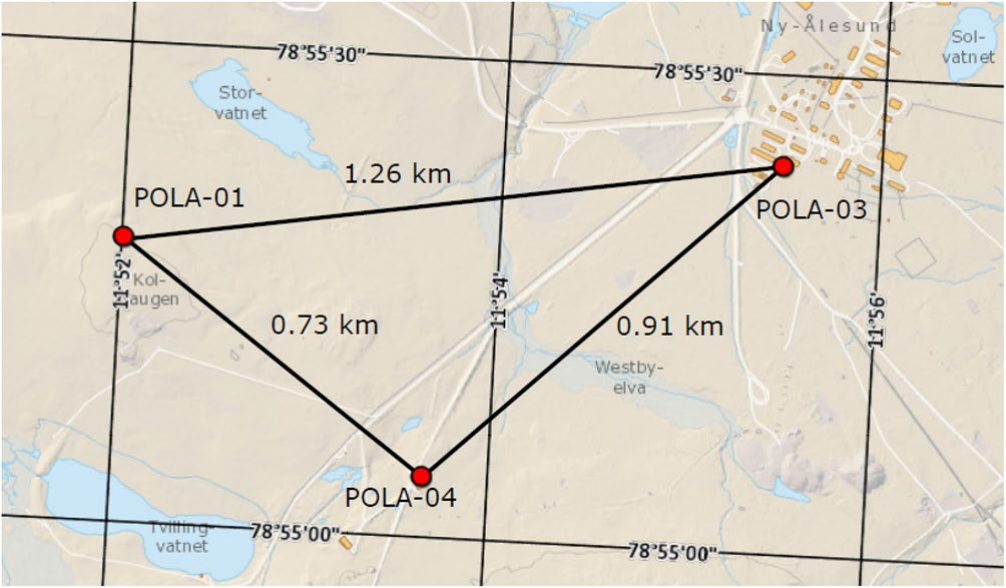
Map of the location of the POLA detectors at the Ny-Ålesund Research Station, on the Spitsbergen island. Courtesy of the Norwegian Polar Institute, retrieved from https://toposvalbard.npolar.no on date 15 June 2022.

Thereafter, in June 2019, three PolarquEEEst detectors (namely POLA-01, POLA-03 and POLA-04) were moved and installed at the Ny-Ålesund Research Station, on the Spitsbergen island. They are located at the three vertices of a triangle of about 1 km of side, as shown in Fig. 10. The detectors are hosted at the research infrastructures managed by the Consiglio Nazionale delle Ricerche (CNR), namely the Arctic Station “Dirigibile Italia”, the Amundsen-Nobile Climate Change Tower, and Gruvebadet Aerosol laboratory, respectively. They have been almost continuously taking data since June 2019, for a long term measurement of the cosmic charged particle rate.

Each POLA detector consists of two planes of plastic scintillator, 1 cm thick, placed inside a light-tight box. The planes are 10 cm apart and each consists of four $20 \times 30$ cm$^2$ tiles, each readout at opposite corners by two Silicon Photo Multipliers (SiPMs), connected to suitable trigger and data acquisition electronics. The whole system is controlled by a Raspberry Pi 3 B+ board (RPi3)^19^. Details about the POLA detectors and the measurement of the cosmic charged particle rate in the latitude range between 66$^{\circ }$ N and $82^{\circ }07^{\prime }$N cited above are collected in^7^.

Multiple sensors are hosted on the POLA detectors. GPS (Global Positioning System) and GLONASS (GLObal NAvigation Satellite System) boards are used to provide the precise time, the local position and the one pulse-per-second (PPS) signal. A 100 MHz system clock is also used to provide a precise event timestamp, together with the PPS.

Several other sensor modules, connected to the RPi3, are used to monitor the temperature, the pressure and the orientation. In particular, three DS18B20 1-Wire digital thermometer sensors^20^ are used to monitor the temperature inside the electronics box, inside the detector box, and outside. A Sense Hat board, made especially for the Astro Pi mission^21^, is used to monitor the orientation (yaw, pitch, roll) of the device via an accelerometer, a 3D gyroscope, and a magnetometer with the LSM9DS1 sensor^22^, the pressure with the LPS25H sensor^10^, and the humidity with the HTS221 sensor^23^.

The sensing element of the LPS25H pressure sensor consists of a suspended membrane realized inside a single mono-silicon substrate. It is capable of detecting the absolute pressure, since its package is holed to holed to allow external pressure to reach the sensing element. It can measure pressures between 260 and 1260 hPa, with a manufacturer declared precision of 1 Pa RMS, and can be operated over a temperature range extending from −30 $^{\circ }$C to +105 $^{\circ }$C. It is equipped with a I2C interface able to take the information from the sensing element and to provide a digital signal to the external world which, in our case, is directly readout by the RPi3. Its performance are fully suitable for the purposes described in this paper.

For redundancy, a BME280 sensor^11,12^ is also used to monitor the atmospheric pressure, temperature and humidity. The BME280 was especially developed for mobile applications, where size and low power consumption important parameters to be taken into account. Its pressure sensor is an absolute barometric pressure sensor, able to measure pressures in the range between 300 and 1100 hPa, with an absolute pressure accuracy, declared by the manufacturer, between $\pm 1.0$ and $\pm 1.7$ hPa depending on the temperature and pressure ranges of operation.

All these sensors are readout every 30 s and, if not otherwise specified, the plots in this paper will have this time sampling.

#### Filter analysis

For this analysis, a second order Butterworth filter was used and applied to the experimental pressure raw data. Assuming that the pressure shock-wave lasted, roughly, less than 2 h (7200 s), and more than 15 min (900 s), the filter was used, at first, as a high pass filter, to cut off frequencies lower than $f_{low}=1/7200$ Hz, then, subsequently, as a low-pass filter, to cut frequencies higher than $f_{high}=1/900$ Hz, similarly to what was done in Ref.^4^. The function signal.butter of the scipy Python library was used to filter the pressure data^24^. The results obtained applying this filter to the data are shown and discussed in the following Section 2.

#### Search for local excesses

In order to search for possible local excesses in the pressure values recorded by the POLA detectors we also implemented a $\chi ^2$ fitting procedure. Basically, fits are performed in sliding time windows (see for instance Refs.^25,26^ and references therein) to search for local Gaussian-like signature on top of a smooth second-order polynomial function. Each pressure window is defined as the interval $[t_w - w, t_w + w]$, where $t_w$ is the time corresponding to the center of the window. The parameter *w* is chosen in order to ensure that the width of the windows is larger than the time span corresponding to the pressure variations due to the shock wave. In the present analysis we used $w=1$ h, and the time window slides across the whole period of data taking in 30 s steps.

In this method we assume a pressure model *P*(*t*) as the sum of a continuous smooth component $P_{s}(t)$ with a possible additional feature $P_f(t)$, i.e. $P(t) = P_{s}(t) + P_f(t)$. The continuous term $P_{s}(t)$ can be described, in the 2-h time window, as a second degree polynomial $P_{s}(t; a_0, a_1, a_2, t_w) = a_0 + a_1 (t-t_w) + a_2 (t-t_w)^2$, where $a_i$ are parameters to be calculated with a best fit procedure. Thereafter, we assume a Gaussian-like function centered on the pressure window to describe the temporal feature: $P_f(t)= \Delta P ~ \exp ( -0.5 (t-t_w)^2/\sigma ^2 )$, where $\Delta P$ represents the pressure amplitude of a possible positive/negative feature with respect to the smooth baseline, and $\sigma$ is related to the time duration of the feature. In the current analysis we used different values for $\sigma$, namely 3, 5, 8, 10, 15 and 20 min.

We define the $\chi ^2$ function in a time window as:

2 $$\begin{aligned} \chi ^2 = \sum _{t_w-w< t_i <t_w+w} \frac{(P_i - P(t_i))^2}{\sigma _i^2} \end{aligned}$$

where $P_i$ is the measured pressure value and $P(t_i)$ is the value of the pressure model defined above calculated at the time $t_i$. $\sigma _i$ is the uncertainty in the $i-th$ measurement, assumed to be 0.1 hPa.

Goal of this analysis is to test whether a signal feature is significantly observed in the pressure data, therefore we tested the hypothesis of pressure variation signal against the null hypothesis in which no pressure feature signals are included. In the null hypothesis model ($H_0$) we include only the smooth model $P_{s}(t)$. In the alternative hypothesis model ($H_1$) we also include the pressure feature $P_f(t)$ in the model.

In each pressure fit window we evaluated the local test statistic $\Delta \chi ^2 = \chi ^2_{0,min} - \chi ^2_{1,min}$, where $\chi ^2_{0,min}$ and $\chi ^2_{1,min}$ are the $\chi ^2$ values corresponding to the best fits (namely, the minimum $\chi ^2$ obtained varying the parameters of the fit functions) of the models corresponding to the null hypothesis and the alternative hypothesis, respectively.

Since the best fit procedure is applied to several pressure time windows partially overlapping to each other, and therefore the same pressure values are actually used many times, the $\Delta \chi ^2$ values obtained in this way are not independent. The degree of correlation depends on the width of the fitted time window and on the sigma of the Gaussian-like feature. In addition, local pressure fluctuations are also expected, that could mimic any anomalies in the observed values. To account for systematic uncertainties that may induce a false signal, or mask a true signal in our fitting procedure, we applied the very same analysis chain described above also to the data collected in the two weeks before the volcano eruption (control region), to obtain the relative $\Delta \chi ^2$ distribution. Then we computed its quantiles and transformed them in a global significance $\sigma _{global}$ parameter, assuming a normal distribution. The ratio between $\Delta \chi ^2$ measured in the signal region and $\sigma _{global}$ gives an estimate of the probability that the observed pressure feature is just a random fluctuation of the baseline or not.

Note that, since we observed a slightly larger noise in the two weeks of the control region with respect to other periods, the global significance computed in this way is conservative, i.e. the corresponding $\sigma _{global}$ values are probably slightly overestimated and, consequently, the significance of the features observed in the signal region are, correspondingly, underestimated.

## Data Availability

Some plots from data are available at the link https://iatw.cnaf.infn.it/eee/monitor/, by selecting the POLA-01, POLA-03 and POLA-04 detectors. Raw data are available for a reasonable request to the corresponding authors. **Software: ** ROOT https://root.cern/, Python https://www.python.org/, Scipy https://docs.scipy.org/doc/scipy/index.html, NumPy https://numpy.org/doc/stable/index.html, Matplotlib https://matplotlib.org/, Adafruit Industries https://github.com/adafruit, Sense HAT https://pythonhosted.org/sense-hat/.
